# Antibiotic Resistance and Biofilm-Forming Ability in Enterococcal Isolates from Red Meat and Poultry Preparations

**DOI:** 10.3390/pathogens9121021

**Published:** 2020-12-03

**Authors:** Ana Castaño-Arriba, Camino González-Machado, Gilberto Igrejas, Patrícia Poeta, Carlos Alonso-Calleja, Rosa Capita

**Affiliations:** 1 Institute of Food Science and Technology, University of León, E-24071 León, Spain; acasa@unileon.es (A.C.-A.); mgonm@unileon.es (C.G.-M.); carlos.alonso.calleja@unileon.es (C.A.-C.); 2 Department of Food Hygiene and Technology, Veterinary Faculty, University of León, E-24071 León, Spain; 3 Department of Genetics and Biotechnology, University of Trás-os-Montes and Alto Douro, 5000-911 Vila Real, Portugal; gigrejas@utad.pt; 4 Functional Genomics and Proteomics Unit, University of Trás-os-Montes and Alto Douro (UTAD), 5000-811 Vila Real, Portugal; 5 Associated Laboratory for Green Chemistry (LAQV-REQUIMTE), University NOVA of Lisboa, 1099-085 Lisboa, Portugal; ppoeta@utad.pt; 6 Microbiology and Antibiotic Resistance Team (MicroART), Department of Veterinary Sciences, University of Trás-os-Montes and Alto Douro (UTAD), 5000-911 Vila Real, Portugal

**Keywords:** antibiotic resistance, biofilm, enterococci, meat preparations

## Abstract

This study investigated the resistance to antibiotics and the capacity to form a biofilm of 200 isolates of enterococci isolated from raw preparations of beef (51 strains), pork (47), chicken (50), and turkey (52) acquired in north-western Spain. Fifteen antimicrobials of clinical importance were tested by the disc diffusion method. The average number of resistances per strain was 4.48 ± 1.59. If resistant strains were taken together with those showing reduced susceptibility, the total number of resistances per strain was 6.97 ± 2.02. Two isolates (1.0% of strains) were resistant to a single antibiotic, twenty-two isolates (11.0%) presented resistance to two, one strain (0.5%) was resistant to three, and 175 isolates (87.5%) showed a multiple drug-resistant phenotype (MDR; defined as no susceptibility to at least one agent from each of three or more antimicrobial categories). The prevalence of resistance varied between 0.5% (gentamicin) and 100% (kanamycin). All strains produced biofilm on polystyrene microwell plates, determined using crystal violet assay. Isolates were classified as having a weak (51 strains; average optical density at 580 nanometers -OD_580_- = 0.206 ± 0.033), moderate (78 strains; average OD_580_ = 0.374 ± 0.068), or strong (71 strains; average OD_580_ = 1.167 ± 0.621) ability to produce biofilm (*p* < 0.05). Isolates from beef preparations produced the most substantial (*p* < 0.05) biofilms. The results of this study indicate that meat and poultry preparations are major reservoirs of antibiotic-resistant enterococcal strains capable of forming a biofilm. In order for food-borne infections to be prevented, the importance of careful handling of these foodstuffs during preparation, avoiding cross-contamination, and ensuring thorough cooking, is stressed.

## 1. Introduction

Meat production worldwide in 2017 reached 334.2 million tons, of which 119.9 million tons were pork, 109.1 million tons poultry, and 66.3 million tons beef. In the European Union (EU), 23.7 million tons of pork, 12.0 million tons of poultry, and 7.8 million tons of beef were produced. Consumption of the three most frequently eaten types of meat in the EU in 2013 was 39.0 kg of pork, 22.5 kg of poultry (mostly chicken), and 12.2 kg of beef per person per year [1]. Some part of this meat is consumed in the form of meat preparations. In accordance with Regulation (EC) 853/2004, these are defined as fresh meat, including meat that has been reduced to fragments, which has had foodstuffs, seasonings, or additives added to it or has undergone processes insufficient to modify the internal muscle fiber structure of the meat and thus eliminate the characteristics of fresh meat [2].

Recent decades have seen increasing preoccupation with antibiotic-resistant bacteria, which are currently considered one of the main problems for health systems all around the world [3]. The prevalence of resistance to antibiotics is on the rise, with it being estimated that within thirty years, there will be yearly deaths in the order of ten million people worldwide as an outcome of infection by resistant bacteria (a larger figure than the 8.2 million deaths expected from cancer). This high rate contrasts with the 700,000 deaths attributable to antibiotic resistance in 2014 [4]. There are also major financial consequences from resistance to antibiotics, with estimates that these infections cost the health-care systems of EU and EEA countries 1.1 million euro annually [5].

The presence of antibiotic-resistant bacteria in foodstuffs implies both direct and indirect risks to consumers. The direct risk is an outcome of the possibility that such microorganisms may cause hard-to-treat foodborne infections, as a result either of insufficient cooking or cross-contamination from other foods through inappropriate handling. The indirect risk lies in the chance of horizontal transfer of resistance genes to pathogenic microorganisms, including unrelated genera [3].

In recent decades, enterococci have emerged as important causes of nosocomial- and community-acquired infections because of the capacity of these bacteria to acquire virulence traits [6]. Moreover, these bacteria act as a reservoir of antibiotic-resistance genes, a circumstance that allows this microbial group to be used as a sentinel for resistance [7]. Monitoring of resistance to antibiotics is essential, not just in obtaining information about the magnitude of the problem and trends within it, but also in planning and providing follow-up for the effectiveness of any control measures introduced.

In nature, microorganisms by preference grow in the form of biofilms, complex communities of microbes embedded in an extracellular polymer matrix synthesized by the organisms themselves, with an ability to adhere to a variety of different biotic or abiotic surfaces [8,9]. Sessile cells that form part of a biofilm have a much-enhanced resistance to different stress factors, such as disinfectants and antibiotics, when compared with planktonic, or free-living, cells. Hence, biofilms have been identified as a determining factor in the persistence of foodborne pathogens in food-processing environments [10]. Biofilms have been shown to be the main source of contamination of foodstuffs and have been associated with the majority of outbreaks of foodborne illness [11]. In addition, serious engineering problems like the obstruction of filters and tubing, or decreased efficiency of heat exchangers, also arise owing to the presence of biofilms on the equipment and in the installations of food-processing plants [8].

There is very little information available about resistance to antibiotics and the capacity to form a biofilm of enterococcal strains found in meat. The aim of the present research was to gain an insight into the patterns of antibiotic resistance and the biofilm-forming ability of enterococci from meat and poultry preparations acquired in north-western Spain.

## 2. Results and Discussion

### 2.1. Microbial Counts

Psychrotrophic counts (log_10_ cfu/g) were 5.88 ± 1.09 in beef preparations, 5.50 ± 2.00 in pork, 5.14 ± 0.89 in chicken, and 6.27 ± 1.17 in turkey (*p* > 0.05). These results fall within the wide range of values (between 3.68 ± 1.75 log_10_ cfu/g and 9.57 ± 0.37 log_10_ cfu/g) previously observed in meat preparations from birds and mammals [12,13]. The samples exceeded the maximum guideline limit of 5 log_10_ cfu/g set in Spain by Pascual-Anderson [14] for dressed poultry.

No signs of deterioration were present to the senses by any of the samples examined, even though in some instances counts for psychrotrophs were above 8 log_10_ cfu/g. This outcome coincides with earlier findings [12]. In contrast, other researchers [15] noted that levels of this microbial group between 6 and 8 log_10_ cfu/g were enough to affect the odor and appearance of meat.

Every sample tested was contaminated with enterococci. The enterococcal loads in the chicken preparations (3.11 ± 1.84 log_10_ cfu/g) were higher (*p* < 0.05) than those observed in beef (1.46 ± 0.72 log_10_ cfu/g), pork (1.87 ± 1.18 log_10_ cfu/g), and turkey (1.91 ± 0.49 log_10_ cfu/g) preparations. The values in question are similar to those observed previously in samples of red meat and poultry preparations, where levels between 0.70 ± 00 log_10_ cfu/g and 3.97 ± 1.29 log_10_ cfu/g were found [12,13].

Determination of the enterococci in meat and meat products is of interest because these microorganisms act as indicators of microbiological quality and standards of hygiene during production and handling, as well as the maintenance of the integrity of the cold chain. In addition, their levels aid in predicting the potential useful life of foodstuffs [16,17]. The presence of a high concentration of enterococci in food products is associated with clearly inadequate hygiene practices, in view of the considerable resistance of these bacteria to drying-out, extreme temperatures, cleaning and disinfecting agents, along with other stress factors [18,19]. Apart from their role as indicator microorganisms, enterococci are responsible for infections and generally present multiple resistances to antibiotics of clinical importance [6,20,21]. Indeed, enterococci are seen as sentinel microorganisms for antibiotic resistance [3].

### 2.2. Antimicrobial Susceptibility

Susceptibility to fifteen antimicrobials of human and veterinary clinical significance was investigated in 200 enterococcal isolates from beef (51 strains), pork (47), chicken (50), and turkey (52) preparations. All the strains presented intermediate susceptibility to at least one antibiotic. An average of 4.48 ± 1.59 resistances per strain was observed. The number of resistances was 4.57 ± 1.37 for isolates from beef, 4.17 ± 1.81 for those from pork, 4.50 ± 1.64 for chicken, and 4.65 ± 1.53 for turkey (*p* > 0.05). If resistance and reduced susceptibility are taken together, the number of resistances per strain was 6.97 ± 2.02. The figure for beef was 7.67 ± 1.51, for pork 6.87 ± 2.33, for chicken 6.42 ± 1.93, and for turkey 6.90 ± 2.11 (*p* > 0.05). Such values are similar to those previously observed with regard to Gram-positive bacteria of meat origin. These were resistant to an average of 6.35 antimicrobials in the case of *Staphylococcus aureus* [12] and of 5.58 for enterococci [21].

Table 1 shows the various phenotypes of resistance, detected by the disc diffusion method, that were found in the 200 strains of enterococci isolated from meat and poultry preparations. A standard definition for acquired resistance to antimicrobials as a way of properly describing multi-drug-resistant (MDR) profiles of bacterial isolates of public health significance has been proposed by a group of international experts working on a joint initiative of the European Centre for Disease Prevention and Control (ECDC) and the Centers for Disease Control and Prevention (CDC), as noted by Magiorakos et al. [22]. These experts defined MDR as acquired non-susceptibility to at least one agent from each of three or more antimicrobial categories (one or more of which must be administered in clinical practice). This criterion was used in the present study to characterize the antibiotic resistance profiles of enterococcal isolates.

None of the strains of enterococci studied was pan-susceptible (susceptible to all antimicrobials tested), whilst two isolates (1.0% of the strains; one isolate from chicken and one from turkey) were resistant to just one antibiotic. However, twenty-two isolates (11.0%) were resistant to two antibiotics, one (0.5%) was resistant to three antibiotics, and 175 isolates (87.5%) showed a multiple drug-resistant phenotype (MDR). The contamination of red meat and poultry with bacteria resistant to antibiotics is a frequent finding [13,23,24,25,26,27,28,29]. Just as in the present investigation, other authors have observed that 100% of strains from meat showed resistance to one or more antibiotics [30]. Stress must be laid on the large percentage of MDR strains seen in this investigation, as it is higher than most of the figures noted for bacteria isolated from foodstuffs [31], which is an alarming result.

**Figure 1:**
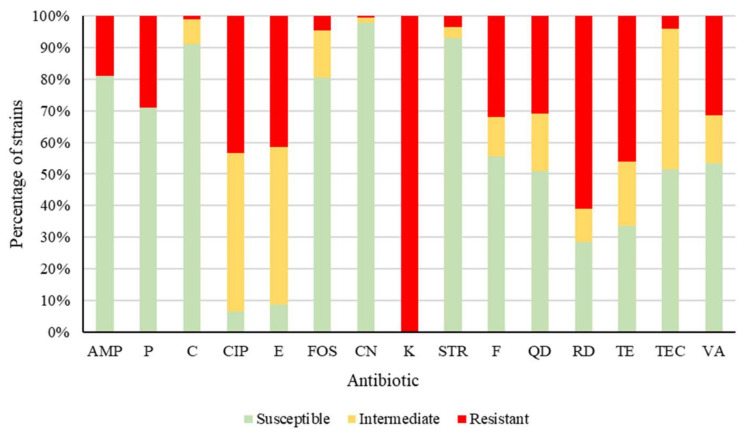
Percentage of enterococci susceptible, intermediate, or resistant to each antibiotic tested. Ampicillin (AMP), penicillin G (P), chloramphenicol (C), ciprofloxacin (CIP), erythromycin (E), fosfomycin (FOS), gentamicin (CN), kanamycin (K), streptomycin (STR), nitrofurantoin (F), quinupristin-dalfopristin (QD), rifampicin (RD), tetracycline (TE), teicoplanin (TEC), and vancomycin (VA).

Figure 1 shows the percentages of strains that were susceptible, intermediate, or resistant to each of the antibiotics tested. No substantial differences were observed between types of meat preparations with regard to the classes of antibiotics to which the strains were resistant. Resistance was observed in enterococcal isolates in respect of ampicillin (AMP), this affected 19.0% of strains, penicillin G (P) at 29.0%, and chloramphenicol (C), with 1.0%. With regard to ciprofloxacin (CIP), the figure was 43.5%, and for erythromycin (E) it amounted to 41.5%. For fosfomycin (FOS), the value was 4.5%, and for gentamicin (CN) 0.5%, but for kanamycin (K) it was 100%. In the case of streptomycin (STR), the figure was 3.5%, and for nitrofurantoin (F) it was 32.0%, while quinupristin-dalfopristin (QD) showed 31.0%. The value for rifampicin (RD) was 61.0%, for tetracycline (TE) 46.0%, for teicoplanin (TEC) 4.0%, and for vancomycin (VA) 31.5%. Resistance or reduced susceptibility was observed for AMP (19.0% of strains), P (29.0%), C (9.0%), CIP (93.5%), and (91.5%), FOS (19.5%), CN (2.0%), K (100%), STR (7.0%), F (44.5%), QD (49.0%), RD (71.5%), TE (66.5%), TEC (48.5%), and VA (46.5%). Resistance to these antimicrobials has previously been reported in enterococcal strains from meat [6,20,21], and this was also observed by other researchers [32,33,34,35,36,37].

The considerable number of bacteria resistant to antibiotics in foodstuffs of animal origin that was recorded in most of the publications consulted is related to the use of antibiotics in various spheres, such as agriculture, animal production, and clinical practice. This has had a great impact on microbial populations and has triggered the selection and proliferation of resistant bacteria [13]. On these lines, the present work observed a great prevalence of resistance to antibiotics widely employed in animal production [38,39,40]. Furthermore, it found a considerable presence of resistance to substances whose use has been prohibited for decades in food-producing animals (for instance, chloramphenicol or nitrofurantoin). It is worth noting that mechanisms for cross-resistance or co-resistance may have contributed to the persistence over time of genes for resistance to these substances, as has previously been suggested [25,41].

The strong prevalence of strains of enterococci that are resistant, or have reduced susceptibility, to various antibiotics that was encountered in this study is a worrying fact. This is because, in the case of infection, there would probably be a downgrading of the usefulness of numerous antibiotics employed in clinical practice in both human and veterinary medicine. In such a scenario, it must be pointed out that ampicillin, ciprofloxacin, erythromycin, fosfomycin, gentamicin, kanamycin, streptomycin, rifampicin, teicoplanin, and vancomycin are all classified as “critically important antimicrobials”, while penicillin G, chloramphenicol, quinupristin-dalfopristin, and tetracycline are categorized as “highly important antimicrobials”, and nitrofurantoin as an “important antimicrobial” for human medicine [42]. According to the World Organization for Animal Health, ampicillin, ciprofloxacin, erythromycin, gentamicin, kanamycin, streptomycin, and tetracycline are classified as “veterinary critically important antimicrobial agents”, while fosfomycin and rifampicin are considered as “veterinary highly important antimicrobial agents” [43].

### 2.3. Biofilm-Forming Ability

**Figure 2:**
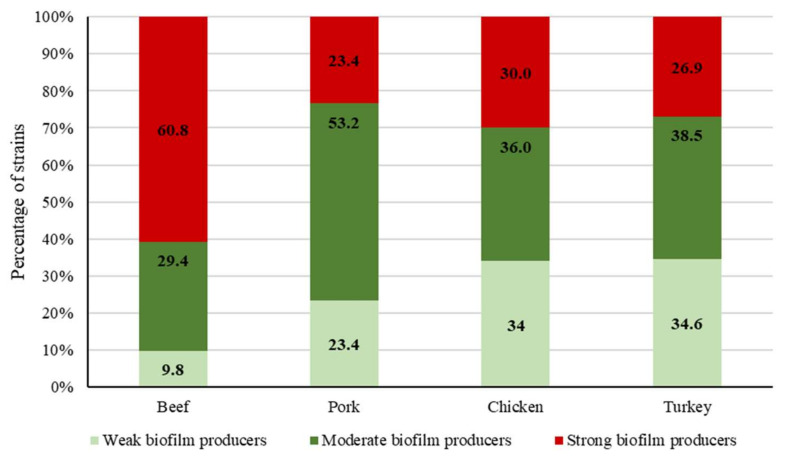
Percentages of weak, moderate, and strong biofilm-producing enterococci found in beef, pork, chicken, and turkey preparations.

The capacity of bacteria to form biofilm is a matter of concern both in the food industry and in clinical settings [8]. In the present research, all the strains of enterococci studied produced biofilm on polystyrene microwell plates, with OD_580_ values (crystal violet assay) ranging from 0.146 ± 0.012 to 3.192 ± 0.024. The average OD_580_ observed among the 200 strains tested was 0.613 ± 0.558. A cut-off value of three standard deviations above the mean OD_580_ of the negative controls, calculated at 0.133, was used for strain classification. Isolates were classified as weak (51 strains; average OD_580_ = 0.206 ± 0.033), moderate (78 strains; average OD_580_ = 0.374 ± 0.068), and strong (71 strains; average OD_580_ = 1.167 ± 0.621) producers of biofilm (*p* < 0.05). While the ability of enterococci to produce biofilm on polystyrene has been previously demonstrated, it must be pointed out that the percentage of biofilm-forming strains of enterococci observed in the present investigation (100%) is higher than the figures obtained from foodstuffs, both in previous studies [6] and in research work by other authors [44,45,46,47]. Figure 2 shows the percentage of strong, moderate, and weak biofilm producers in isolates from beef, pork, chicken, and turkey. Beef samples showed the highest (*p* < 0.05) percentage of strong biofilm-producing strains.

One noteworthy point is that the strains that were strong producers of biofilm showed a higher (*p* < 0.05) average number of resistances (4.73 ± 1.49) than the weak producers (4.06 ± 1.74). The average number of resistances per strain in moderate biofilm producers was 4.53 ± 1.54 (*p* > 0.05). This is the first time that a direct relationship between antibiotic resistance and biofilm formation has been reported in enterococci from meat and poultry preparations, and further studies are needed to support this finding. Cepas et al. [48] have demonstrated that the acquisition of specific antimicrobial resistance can compromise or enhance biofilm formation in several species of Gram-negative bacteria. By contrast, other authors have observed that isolates with a higher level of resistance tended to form weaker biofilms [49]. It has been suggested that biofilm acts as a mechanism for bacteria to get a better survival, especially in isolates with resistance levels not high enough. Moreover, even though biofilms formed by isolates with a high level of resistance are weak, they could still provide a similar level of protection for the isolates.

## 3. Materials and Methods

### 3.1. Samples

Forty-four samples, each weighing approximately 250 g, of raw meat and poultry preparations were acquired from various supermarkets in the city of León, in north-western Spain. With respect to beef, ten samples of hamburgers were obtained. The pork preparations included meatballs (2 samples), minced meat (6), hamburgers (2), and sausages (4). The chicken samples comprised hamburgers (4), nuggets (2), and sausages (2), and the turkey preparations were meatballs (4) and hamburgers (8). All samples were individually placed in sterile plastic bags, transported to the laboratory in an ice chest, and analyzed within a maximum lapse of four hours from collection. Samples were stored at 4 °C until the analysis was performed.

### 3.2. Microbiological Analysis

Portions of 10 g were taken from each sample and placed in sterile stomacher bags together with 90 mL of 0.1% (wt/vol) peptone water (Oxoid Ltd., Basingstoke, England), and then homogenized using a Masticator (IUL, Barcelona, Spain) for two minutes. The 10 g of sample were taken from the various pieces in the same lot. Using the homogenized portions, serial decimal dilutions were produced in the same diluent. Psychrotrophs were determined by means of the spread-plate technique (0.1 mL) on plate count agar (PCA; Oxoid) after ten days of incubation at 7 °C. To enumerate enterococci, duplicate pour plates of kanamycin aesculin azide (KAA; Oxoid) agar, prepared using 1 mL volumes of appropriate dilutions, were incubated for twenty-four hours at 42 °C. The plates with between 25 and 250 colonies (spread-plate technique) and between 30 and 300 colonies (pour-plate technique) were counted, and the mean counts calculated and transformed to log_10_ colony-forming units per gram (cfu/g). From each sample, between 3 and 7 typical colonies on KAA were selected. These colonies were transferred onto tryptone soy agar (TSA; Oxoid) and incubated at 42 °C for 24 h to obtain pure cultures. Gram-positive, catalase-negative cocci able to grow at 10 °C and 45 °C, and in the presence of 6.5% NaCl were tested for antimicrobial susceptibility and biofilm-forming ability. The strains were kept frozen at −50 °C after re-suspension in tryptone soy broth (TSB; Oxoid) with 20% (vol/vol) glycerol.

### 3.3. Antimicrobial Susceptibility Testing

The susceptibility of 200 enterococcal isolates to a panel of fifteen antibiotics was determined using the disc diffusion method [50], using antibiotic discs (Oxoid) on Mueller Hinton agar (Oxoid). The following antibiotic classes were tested: penicillins (ampicillin -AMP, 10 μg-, penicillin G -P, 10 μg-), amphenicols (chloramphenicol -C, 30 μg-), fluoroquinolones (ciprofloxacin -CIP, 5 μg-), macrolides (erythromycin -E, 15 μg-), phosphonic acid derivatives (fosfomycin -FOS, 50 μg-), aminoglycosides (gentamicin -CN, 120 μg-, kanamycin -K, 120 μg-, streptomycin -STR, 300 μg-), nitrofuran derivatives (nitrofurantoin -F, 300 μg-), streptogramins (quinupristin-dalfopristin -QD, 15 μg-), ansamycins (rifampicin -RD, 5 μg-), tetracyclines (tetracycline -TE, 30 μg-), and glycopeptides (teicoplanin -TEC, 30 μg-, vancomycin -VA, 30 μg-). These antibiotics are classified as critically important (AMP, CIP, E, FOS, CN, K, STR, RD, TEC, VA), highly important (P, C, QD, TE), or important (F) for human medicine [42]. According to the World Organization for Animal Health, such compounds are categorized as veterinary critically important (AMP, CIP, E, CN, K, STR, TE) or veterinary highly important (FOS, RD) antimicrobial agents [43]. Inhibition haloes were measured after incubation at 37 °C for 18 to 24 h, and isolates were classified as susceptible, intermediate (reduced susceptibility), or resistant. *Escherichia coli* ATCC 25922 and *Staphylococcus aureus* ATCC 29213 were used as reference strains for antibiotic disc control.

### 3.4. Biofilm Determination

In studying biofilms, a previously described procedure [8] was followed. The 200 strains investigated, grown on TSA, were transferred to test-tubes of TSB and incubated at 37 °C. After 18 h, the concentration of bacteria in the tubes was approximately 10^9^ cfu/mL (data not shown). Four decimal dilutions in TSB were carried out so as to obtain concentrations of 10^5^ cfu/mL, these being used to inoculate the wells of polystyrene microwell plates (Oy Growth Curves Ab Ltd., Helsinki, Finland). The wells were filled with 225 µL of TSB and 25 µL of bacterial culture, so that the final concentration in the wells was 10^4^ cfu/mL. Negative controls with 250 µL of TSB were employed. After incubation at 37 °C for 24 h, the contents of the plates were poured off and the wells washed with 300 µL of sterilized distilled water. Thereafter, 250 µL of methanol was added to each well and allowed to act for 15 min. Once this time had elapsed, the plates were emptied, air-dried, and stained by the addition of 250 µL of an aqueous solution of 0.5% crystal violet to each well. After five minutes, the wells were emptied and rinsed by putting the plates under running water from the tap. The plates were then air-dried once more, and the dye bound to the adherent cells was re-solubilized using 250 µL of 33% acetic acid (Sigma-Aldrich Co., St. Louis, MO, USA) per well. Sixty seconds of contact was allowed, then the optical density at 580 nm (OD_580_) was determined in a Bioscreen C MBR (Oy Growth Curves Ab). The microwell plates were agitated for one minute prior to the measurement of turbidity. In each experiment, strong and weak biofilm-forming strains from the culture collection of the Department of Food Hygiene and Technology of the University of Leon in Spain were included.

To classify strains as a function of their capacity to form a biofilm, the cut-off OD_580_ (ODc) was calculated, this being set at a value of three standard deviations above the mean OD_580_ of the negative controls. Strains were grouped into four categories [8]. The first comprised those not considered biofilm producers when OD_580_ ≤ ODc. The other three were weak biofilm producers when ODc < OD_580_ ≤ (2 × ODc), moderate biofilm producers when (2 × ODc) < OD_580_ ≤ (4 × ODc), and strong biofilm producers when (4 × ODc) < OD_580_.

### 3.5. Statistical Analysis

Microbial counts in log_10_ cfu/g and OD_580_ values from the crystal violet assay were examined by analysis of variance (ANOVA) techniques, using Duncan’s multiple range test to separate averages. The prevalence of resistance in the different types of meat was compared using Fisher’s Exact Test. Significance was determined at the 95% level (*p* < 0.05). All the tests were carried out using the Statistica^®^ 8.0 package (Statsoft Ltd., Tulsa, OK, USA).

## 4. Conclusions

The results of this research make it plain that the strains of *Enterococcus* spp. isolated from meat and poultry preparations pose a potential threat for consumers, in view of the considerable prevalence of strains either resistant or with reduced susceptibility to antibiotics. This is a worrying finding from the point of view of public health and food safety, not only because of the direct risk of infection from the consumption or handling of meat preparations contaminated with enterococci but also because of the major indirect risk arising from possible horizontal gene transfer into other pathogenic bacteria. The enterococcal strains studied evinced weak (25.5%), moderate (39.0%), or strong (35.5%) abilities to form biofilm on polystyrene. The results from this research suggest a direct relationship between antibiotic resistance and the capacity to produce biofilm. The necessity of taking measures to reduce the rates of resistance to antibiotics in the bacteria present in meat and poultry preparations, including the prudent use of antibiotics in animal production, is highlighted. The importance of the careful handling of these foodstuffs to avoid cross-contamination and the need to ensure thorough cooking is also underlined.

## Figures and Tables

**Table 1:** Antibiotic-resistance patterns in 200 enterococci isolates from red meat and poultry preparations.

Antibiotic Resistance Pattern	Number of Strains
K	2
CIP/K	2
E/K	6
K/RD	4
K/TE	9
K/VA	1
AMP/P/K	1
AMP/CIP/K	1
P/CIP/K	1
P/K/RD	2
P/K/TE	2
CIP/K/RD	3
CIP/K/TE	2
E/K/QD	1
E/K/TE	8
E/K/VA	1
FOS/K/VA	1
P/K/F	2
K/F/RD	6
K/F/TE	1
K/QD/TE	2
K/RD/VA	1
AMP/P/K/F	1
AMP/P/K/RD	1
AMP/CIP/K/RD	2
AMP/CIP/K/TE	1
AMP/E/K/RD	2
P/CIP/K/F	2
P/CIP/K/RD	1
P/CIP/K/TE	1
P/K/F/RD	3
P/K/RD/TE	1
C/E/K/TE	1
C/K/F/RD	1
CIP/E/K/F	1
CIP/E/K/TE	4
CIP/K/F/RD	3
CIP/K/F/TE	2
CIP/K/F/VA	1
E/K/F/RD	1
E/K/QD/RD	3
E/K/QD/VA	1
K/QD/TE/VA	4
K/QD/RD/VA	5
K/RD/TE/VA	1
AMP/CIP/E/K/RD	1
AMP/P/CIP/K/F	2
AMP/P/CIP/K/ RD	1
AMP/CIP/K/STR/TE	1
AMP/P/CIP/K/F/TE	1
AMP/P/K/F/RD	2
P/CIP/K/F/TE	1
P/CIP/K/F/RD	3
P/E/K/F/RD	1
P/FOS/K/F/RD	1
P/K/F/RD/VA	1
P/E/K/RD/TE	1
CIP/E/K/F/TE	1
CIP/E/K/RD/TE	1
CIP/K/QD/RD/VA	3
CIP/K/F/RD/TE	1
E/K/F/RD/TE	3
E/K/STR/QD/RD	1
E/K/QD/RD/VA	8
E/K/RD/TEC/VA	1
AMP/P/CIP/E/K/TE	1
AMP/P/CIP/K/TE	2
AMP/P/CIP/K/RD/TE	1
AMP/CIP/K/STR/QD/TE	1
AMP/P/CIP/K/F/TE	2
AMP/P/CIP/K/F/RD	1
AMP/P/E/K/F/RD	2
AMP/P/E/K/F/TE	1
P/CIP/E/K/F/TE	1
P/CIP/E/K/RD/TE	1
P/CIP/K/F/RD/TE	1
P/CIP/K/F/RD/VA	1
P/E/FOS/K/F/RD	1
P/E/K/QD/RD/VA	1
CIP/E/K/F/RD/TE	1
CIP/E/K/QD/RD/TE	2
CIP/E/K/QD/RD/VA	4
CIP/E/K/QD/TE/VA	1
CIP/K/QD/RD/TE/VA	6
CIP/E/FOS/K/F/RD	1
E/K/QD/RD/TE/VA	3
E/K/QD/RD/TEC/VA	3
K/QD/RD/TE/VA	5
AMP/CIP/E/FOS/K/F/RD	2
AMP/P/CIP/E/FOS/K/TE	1
AMP/P/CIP/K/F/RD/TE	3
AMP/P/E/FOS/K/F/RD	1
P/CIP/E/K/F/RD/TE	1
P/CIP/E/K/F/RD/VA	1
P/CIP/K/RD/TE/TEC/VA	1
CIP/E/K/QD/RD/TE/VA	1
CIP/K/QD/RD/TE/TEC/VA	2
E/K/STR/F/QD/RD/TE	1
E/K/STR/QD/RD/TE/VA	1
AMP/CIP/E/K/STR/QD/RD/VA	1
AMP/P/CIP/K/F/RD/TE/VA	1
CIP/E/CN/K/QD/RD/TE/VA	1
AMP/P/CIP/E/K/FOS/K/F/RD	1
CIP/E/K/STR/QD/RD/TE/TEC/VA	1

Ampicillin (AMP), penicillin G (P), chloramphenicol (C), ciprofloxacin (CIP), erythromycin (E), fosfomycin (FOS), gentamicin (CN), kanamycin (K), streptomycin (STR), nitrofurantoin (F), quinupristin-dalfopristin (QD), rifampicin (RD), tetracycline (TE), teicoplanin (TEC), and vancomycin (VA).
